# Ofatumumab successfully treats myelin oligodendrocyte glycoprotein antibody-associated disease accompanied by Epstein-Barr viral infection: a case series

**DOI:** 10.3389/fimmu.2024.1510097

**Published:** 2025-01-07

**Authors:** Chen Min, Bi Zhuajin, Liu Peidong, Li Ruoyu, Liu Ju, Liu Hongbo

**Affiliations:** ^1^ Department of Neurology, The First Affiliated Hospital of Zhengzhou University, Zhengzhou, Henan, China; ^2^ Department of Neurosurgery, The First Affiliated Hospital of Zhengzhou University, Zhengzhou, Henan, China

**Keywords:** MOG-IgG associated encephalitis, Epstein-Barr virus, ofatumumab, neuron damage, case report

## Abstract

Myelin oligodendrocyte glycoprotein antibody-associated disease (MOGAD) caused by pathogenic immunoglobulin G antibodies to myelin oligodendrocyte glycoprotein is a rare demyelinating disease of the central nerve system (CNS). The clinical phenotypes of MOGAD include acute disseminated encephalomyelitis, optic neuritis, and transverse myelitis. At present, the mechanism underlying the disease is unknown. Herein, we report two cases of MOGAD accompanied by Epstein-Barr virus (EBV) infection. Both presented inflammation response in the cerebrospinal fluid (CSF), demonstrating elevated level of cell accounts and protein. EBV genomic sequence was also detected in the CSF samples. The patients recovered substantially after 3 months following a combination treatment with methylprednisolone and immunosuppressive therapy with ofatumumab. These cases provide new insight into the production of MOG-IgG and the possible pathological mechanisms underlying MOGAD. The cases also confirm the association with EBV, a virus that infects human B cells and has been proposed to be a trigger for MOGAD. This is the first report on subcutaneous ofatumumab treatment for MOGAD triggered by EBV, suggesting that this is a potentially valuable therapeutic option.

## 1 Introduction

Myelin oligodendrocyte glycoprotein (MOG) is expressed exclusively on the surface of oligodendrocytes in the central nervous system (CNS). Myelin oligodendrocyte glycoprotein antibody- associated disease (MOGAD) is a rare demyelinating disease of the CNS. The clinical presentation of MOGAD is kaleidoscopic, but it often includes encephalomyelitis, optic neuritis, and transverse myelitis. The mechanism of MOG antibody production, as well as that underlying blood-brain barrier (BBB) dysfunction, are unclear at present. Previous infections with Epstein-Barr virus (EBV), which predominantly affects B cells, has been proposed as a trigger for MOGAD. A cohort study conducted by Molazadeh et al. showed that 59.7% patients experienced a relapsing course for the disease (1). Therapy consisting of B cell-depleting monoclonal antibodies was shown to decrease recurrence. Ofatumumab, which bind to CD20+ B cells, has shown efficacy in the treatment of many autoimmune diseases, including a robust therapeutic effect against AEs (2). However, the effect of ofatumumab against MOGAD accompanied by EBV infection remains uncharacterized. Herein, we report two cases of MOGAD with EBV infection that showed quick improvement after receiving three injections of ofatumumab during a follow-up period of approximately 3 months.

## 2 Case description

### 2.1 Case 1

**Figure 1:**
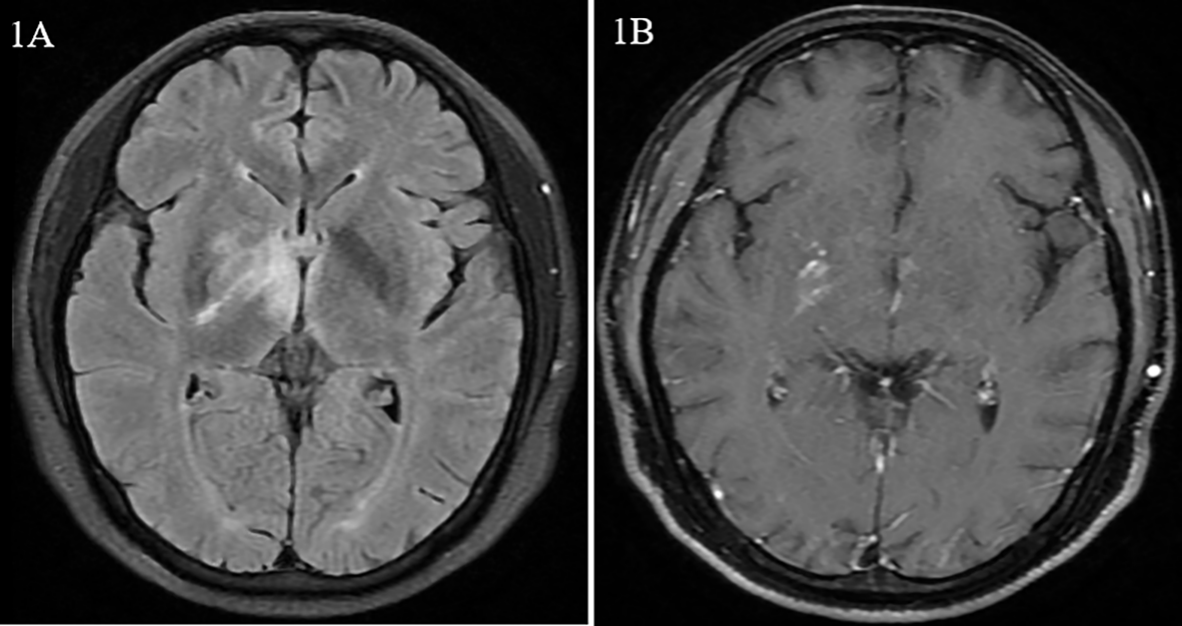
Magnetic resonance images from case 1. **(A)** Hypersignal of the right basal ganglia and thalamus on fluid-attenuated inversion recovery (FLAIR). **(B)** Nodular enhancement of the right cerebral peduncle on dynamic enhanced magnetic resonance imaging.

A 33-year-old male patient was admitted to our department, complaining of fever lasting for 40 days. Forty days prior to admission, the patient had a low fever of 38°C. Cranial plain magnetic resonance imaging (MRI) performed half a month prior to admission revealed hypointensity on T1-weighted images, hyperintensity on T2-weighted images, and hyperintensity on T2-fluid-attenuated inversion recovery (FLAIR) in the right temporal lobe, right pontine, right thalamus, and right basal ganglia with nodular enhanced (**Figures 1A, B**). Antibiotics and antiviral drugs were administered without improvement. The lesions in the brain expanded, and the patient experienced headache and blurred vision. He was otherwise healthy, with no history of similar diseases or any family history of autoimmune diseases.

Physical examination revealed somnolence, failing eyesight (best-corrected visual acuity of 0.9 in both eyes), dementia, and a positive meningeal irritation sign. No signs of recent infection (pharyngitis, hepatitis, mononucleosis syndrome) were observed. In the neuropsychological evaluation, the patient scored 16/30 on the mini-mental state examination (MMSE) after 12 years of education. The IgG antibody to EBV in the serum was positive while the IgM antibody was negative. The EBV DNA tested by PCR was negative in the serum.

**Figure 2:**
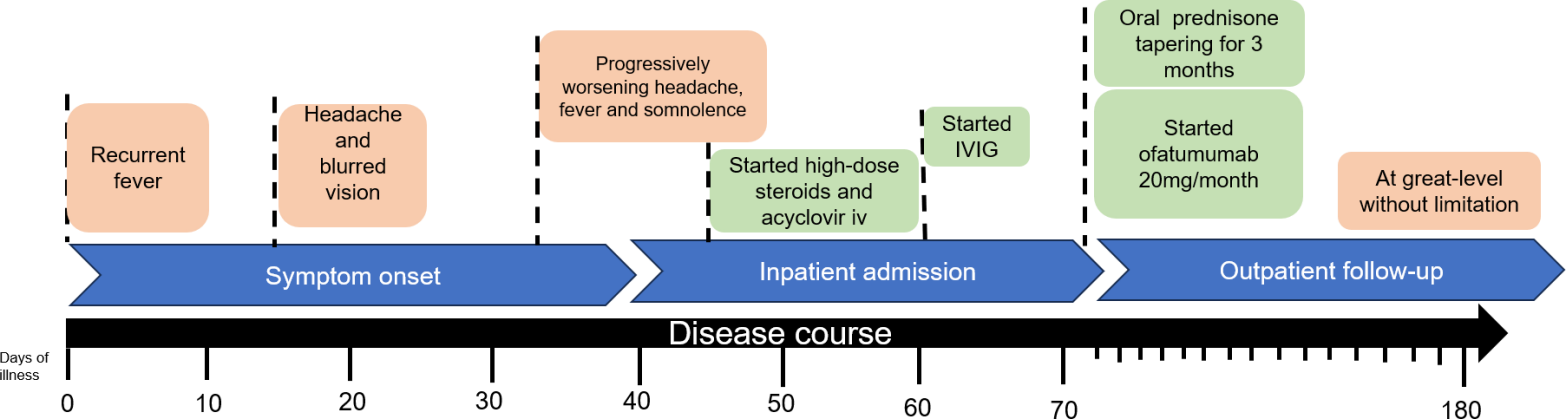
Timeline of clinical course and treatment regimens for case 1.

Cerebrospinal fluid (CSF) examination revealed elevated pressure (200 mmH_2_O, normal range: 80-180 mmH_2_O), pleocytosis (184 cells/mm^3^, normal range <5 cells/mm^3^) with lymphocytic dominance in cytology, elevated protein concentration (612.0 mg/L, normal range:150-450 mg/L), an IgG index of 0.6 (normal value: <0.85), and the absence of oligoclonal IgG bands. Both the IgG and IgM antibodies in the CSF were negative. EBV was detected using next-generation sequencing (sequence number: 1329), and MOG-IgG was detected in the serum (titer: 1:32) and CSF (titer: 1:10) by a cell-based assay (CBA). The serum neurofilament light chain protein level was 40.88 pg/mL (normal range: 0-8.1 pg/mL). No high affinity was observed in 18F- fluorodeoxyglucose-positron emission tomography/computed tomography (PET-CT). Ultimately, he was diagnosed with MOGAD. Acyclovir (0.5 q8h ivgtt) and corticosteroids (500 mg/day for 3 days) were administered; however, the symptoms were not improved, with persistent high-grade fever, severe headache, and lethargy. Subsequent CSF analysis revealed pleocytosis (540/mm^3^), a protein concentration of 1320.0 mg/L, an IgG index of 0.87, and absence of oligoclonal IgG bands. Intravenous immunoglobulin (2 g/kg) was administered for 5 days, combining corticosteroids (250 mg/day for 3 days, 120mg/day for 3 days following oral corticosteroid therapy tapering) and symptoms improved subsequently, with normal temperature, no headache, and no signs of meningeal irritation. The patient scored 28/30 on the MMSE. The adherence was good, and there were no adverse events. Ofatumumab (20 mg/month) combining oral corticosteroid were prescribed for prevention due to the severe clinical presentation and the heavy burden of cranial lesions. During the 6-month follow-up period, the patient achieved complete recovery, and the titer of MOG-IgG in serum decreased from 1:32 to 1:10. The timeline of the treatment is shown in **Figure 2**.

### 2.2 Case 2

**Figure 3:**
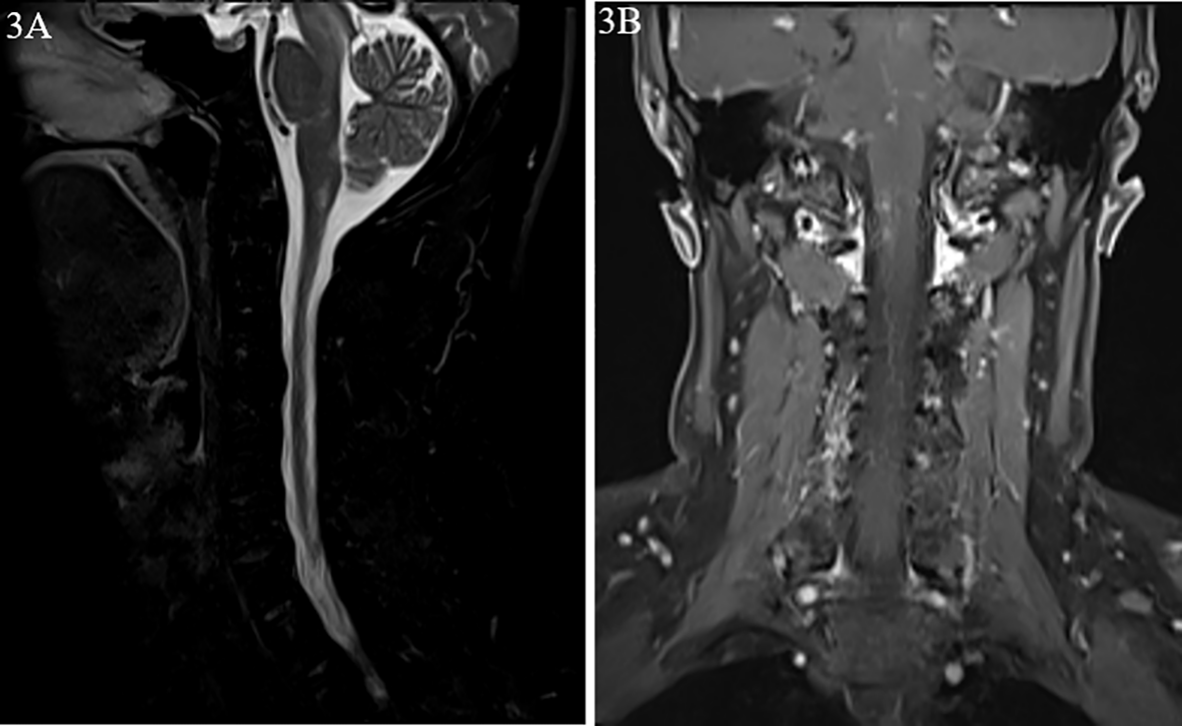
Magnetic resonance images from case 2. **(A)** Hypersignal of the brain stem and the area postrema on T2-weighted image. **(B)** Nodular enhancement on dynamic enhanced magnetic resonance imaging (MRI).

A 45-year-old male patient was admitted to the Department of Gastroenterology of our hospital with the primary complaint of hiccups and low fever (below 37.5°C) for 4 days. His CT scan of the whole abdomen was normal. Baclofen was used for treatment, but no improvement was observed. Moreover, he suffered from diplopia. He was then transferred to the Neurology Department. Cranial and cervical MRI revealed hypointensity on T1-weighted images, and hyperintensity on T2 FLAIR images in the brain stem and the area postrema with enhancement of meninges (**Figures 3A, B**). The patient was healthy, with no history of similar symptoms or family history of autoimmune diseases.

**Figure 4:**
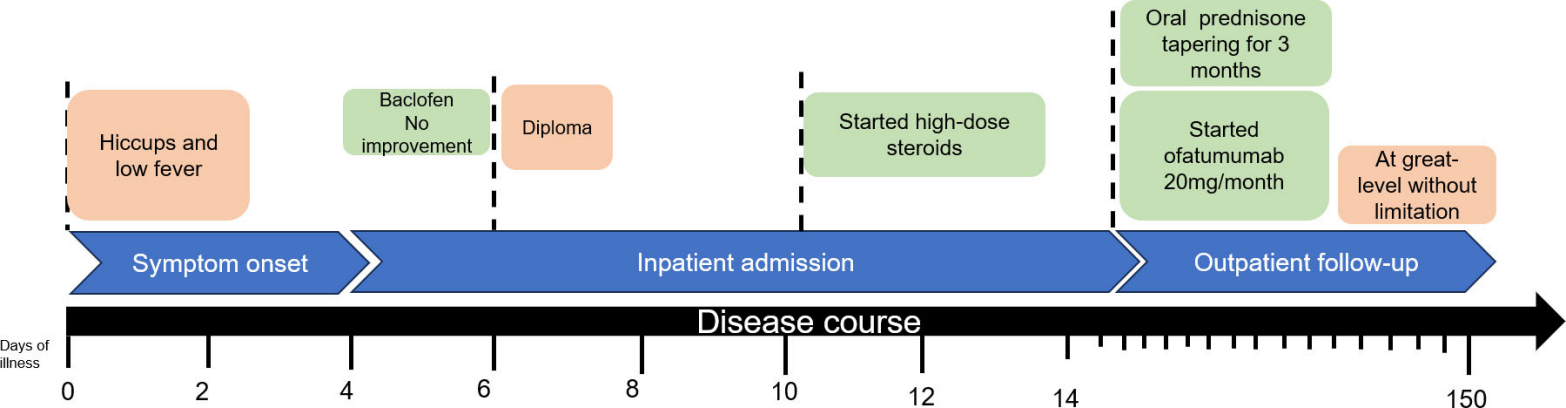
Timeline of clinical course and treatment regimens for case 2.

Physical examination revealed partial oculomotor nerve paralysis. No signs of recent infection (pharyngitis, hepatitis, mononucleosis syndrome) were observed. The IgG antibody to EBV in the serum was positive while the IgM antibody was negative. The EBV DNA tested by PCR was negative in the serum. CSF examination showed normal pressure (120 mmH_2_O), pleocytosis (70 cells/mm^3^) with lymphocytic dominance in cytology, and a protein concentration of 505.1 mg/L. Both the IgG and IgM antibodies in the CSF were negative., and infection with EBV was further confirmed using next-generation sequencing (sequence number: 8). Moreover, MOG-IgG was detected in the CSF (titer: 1:10) and in the serum (titer 1:100) using CBA. Ultimately, he was diagnosed with MOGAD. Corticosteroids were administered (500 mg/day for 3 days, 250 mg/day for 3 days, and 120 mg/day for 3 days), and the patient condition improved quickly, without any adverse events. Ofatumumab was prescribed for prevention due to the pervasive lesions. Ofatumumab (20 mg/month) combining oral corticosteroid were prescribed and the patient exhibited good adherence. Complete recovery was achieved after 3 months, with MOG-IgG being detected in the serum (titer: 1:10) using CBA. The timeline of the treatment is shown in **Figure 4**.

## 3 Discussion

Herein, we present the cases of two patients who developed MOGAD accompanied by EBV infection. Moreover, the patients had good prognosis after ofatumumab treatment. They continue to be followed up regularly.

The clinical spectrum of MOGAD is characterized by optic neuritis, transverse myelitis, and cortical and/or brainstem encephalitis, with a relapsing rate of approximately 50%. An infectious prodrome frequently precedes the initial symptom onset in up to 60% of MOGAD cases (3).

As a target autoantigen in demyelinating disease, MOG-IgG has been clearly established to elicit cellular (4) and humoral (5) immune responses. The pathological mechanism underlying MOGAD is thought to be mediated by CD4+ T cells, with B cells playing an important role (6). Due to the high MOG-IgG positivity rate in the serum, it has been further hypothesized that the MOG antigen may leak into the periphery and be recognized by the immune system. When BBB permeability increases, such as during CNS infection, MOG-IgG enters the CNS, causing demyelination. MOGAD has been reported to occur after acute EBV encephalopathy (7). EBV infection is postulated to cause BBB damage, while activated lymphocytes (including T and B lymphocytes as well as plasma cells) infiltrate the brain. B cells subsequently act as antigen-presenting cells (APC) to present MOG antigens to T cells. Anti-MOG antibodies are subsequently synthesized, causing demyelination through the activation of the complement system and the release of pro-inflammatory cytokines (8). Anti-MOG antibodies have been detected in 20% of the patients with infectious mononucleosis, with no neurological manifestations due to primary EBV infection (9). None of these patients suffering infectious mononucleosis and MOG-IgG developed any clinical sign associated with MOGAD (9), so, MOG-IgG observed may be part from a broad non-specific autoimmune reaction associated with infection. For us, it should be careful to associate this point with a direct risk of MOGAD. The occurrence of autoreactive IgG antibodies during symptomatic primary EBV infection could signal a propensity to develop autoimmune diseases in the future. In the two cases described here, EBV infection in the CSF samples was confirmed by metagenomic next-generation sequencing.

Significant and permanent disability can also occur in MOGAD (10), particularly if the first attack was life-threatening or leaves the patient with severe residuals deficits. Therefore, effective treatment for the syndrome is important. Treatment for MOGAD includes acute attack and maintenance therapy. The current recommendations for therapy are mostly empirical and based on standard protocols for the treatment of neuromyelitis optica spectrum disorders. Acute attacks are typically treated with high-dose intravenous methylprednisolone followed by an oral prednisone taper, which can yield significant recession. However, relapses can occur shortly after withdrawal or rapid tapering of prednisone. Patients that do not respond adequately to steroids are often treated with intravenous immunoglobulins, immunoadsorption and plasma exchange (11). There is no available class-I data on maintenance treatment, and empiric maintenance treatment is generally reserved for relapsing cases or patients with severe residual disability after the acute attack. The most commonly used therapeutic agents are low-dose hormones, rituximab, azathioprine, mycophenolate mofetil and intravenous immunoglobin (12). Different immunological agents, including azathioprine, motemycophenolate and methotrexate, may reduce the risk of relapse in patients with MOGAD with a lower effectiveness.

It has been proved that MOG-IgG has pathogenic potential (13). The location of MOG-IgG production is yet to be fully elucidated, although two potential sources of circulating autoantibodies have been proposed: plasmablasts that emerge from germinal center reactions in secondary lymphoid organs, and long-lived plasma cells located in the bone marrow (14). B cells activated in the secondary lymphoid tissue may also migrate into the intrathecal compartment to undergo clonal expansion and differentiate into plasmablasts, potentially associated with the formation of ectopic tertiary lymphoid structures (15). So, B cell depleting therapy has been proved to prevent relapses effectively. Relapse of MOGAD was defined as being a new clinical attack occurring more than 30 days following onset of a previous attack. Relapses are more common in the first 6 months than later after the first attack. Relapses can occur within 2 months following oral corticosteroid therapy tapering or cessation (16, 17). Some patients have a cluster of early relapses (18).

Studies suggested that rituximab effected in 33% to 100% of MOGAD patients (17, 19–21). In the largest international cohort retrospectively analyzing data from 121 patients, relapse rates on rituximab declined by 37%. After 2 years, 33% were predicted to remain relapse free. Rituximab has shown efficacy in up to two-thirds of patients with MOGAD (22). During maintenance therapy of MOGAD, azathioprine, mycophenolate mofetil, and rituximab are the first-line treatments for adults. Ofatumumab is a human recombinant IgG1 CD20 next-generation monoclonal antibody, with stronger complement-dependent cytotoxicity compared to rituximab in *in vitro* studies and low antigenicity. Rituximab resistance may occur in some patients due to Fc receptor polymorphism, which is another advantage of ofatumumab. Moreover, subcutaneous injection is convenient to use. In the two cases described here, we chose ofatumumab to prevent relapse due to the severe onset of the syndrome, the high burden of the lesions in the brain, and the elimination effect of B cells infected by EBV.

## 4 Conclusion

In this report, we present two cases of MOGAD accompanied by EBV infection in the CSF. Based on this experience, we suggest that patients with MOGAD (particularly those with severe CSF inflammatory responses) should be screened for EBV infection. In addition, ofatumumab should be considered as a treatment option for MOGAD with EBV. However, this is based on the outcomes observed in only two clinical cases. Studies involving a larger number of patients should be carried out to confirm the efficacy and safety of the proposed treatment protocol.

## Data Availability

The original contributions presented in the study are included in the article/supplementary material. Further inquiries can be directed to the corresponding author.
